# Supplementary material for: Distinct architectural requirements for the parS centromeric sequence of the pSM19035 plasmid partition machinery
Source: eLife. 2022 Sep 5;11:e79480. doi: 10.7554/eLife.79480 (PMC9499535; doi:10.7554/eLife.79480)
Supplement: Figure 1—figure supplement 2—source data 1. [file elife-79480-fig1-figsupp2-data1.pdf]

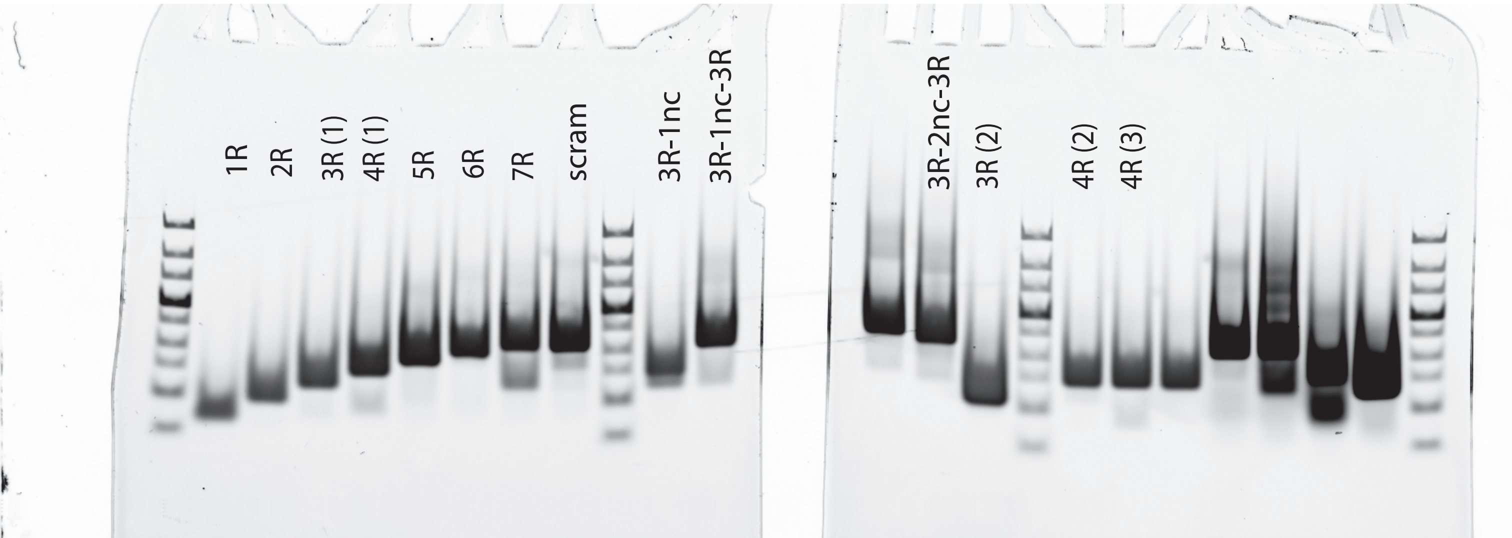
1R
2R
3R (1)
4R (1)
5R
6R
7R
scram
3R-1nc
3R-1nc-3R
3R-2nc-3R
3R (2)
4R (2)
4R (3)
